# Synthesis and Development of Novel Small-Molecule MEIS2 Inhibitors That Induce Cell Death in Breast Cancer Cells by Targeting the Homeobox Domain

**DOI:** 10.3390/ph19060881

**Published:** 2026-06-01

**Authors:** Fatih Kocabaş, Birkan Girgin, Merve Uslu, Pınar Siyah, Arif Mermer

**Affiliations:** 1 Department of Molecular Biology and Genetics, Faculty of Engineering and Natural Sciences, Istanbul Atlas University, 34403 Istanbul, Türkiye; 2 Department of Neuropharmacology, Graduate School of Medicine, Hokkaido University, Sapporo 060-0808, Japan; birkan.girgin.d7@elms.hokudai.ac.jp; 3 Department of Medicine, Johns Hopkins University School of Medicine, Institute for Fundamental Biomedical Research, Johns Hopkins All Children’s Hospital, St. Petersburg, FL 33701, USA; merveuslu9@gmail.com; 4 Department of Biochemistry, School of Pharmacy, Bahcesehir University, 34349 Istanbul, Türkiye; pinar.siyah@med.bau.edu.tr; 5 Faculty of Pharmacy, University of Health Sciences, 34668 Istanbul, Türkiye; arif.mermer@sbu.edu.tr

**Keywords:** MEIS inhibitors, Hox, TALE family, anticancer

## Abstract

**Background**: MEIS proteins are essential homeobox transcription factors that play critical roles in development and have been increasingly implicated in oncogenesis, including breast cancer. **Methods**: In this study, we identified and characterized novel small-molecule MEIS2 inhibitors through in silico docking targeting the active region of the human MEIS2 homeobox domain. Lead candidates MEISi-2E, MEISi-3, and MEISi-4 were identified with binding energies ranging from −3.0 to −3.90 kcal/mol. The inhibitory potential of these molecules was validated in vitro using a species-conserved MEIS-Luciferase Reporter construct containing the TGACAG targeted locus. **Results**: Our results demonstrate that MEISi-2E, MEISi-3, and MEISi-4 significantly suppress MEIS-driven luciferase activity and downregulate the expression of *Meis1*, *Meis2*, and downstream genes such as *IL17RB*, *CDH1*, *EGR2*, *PAX6*, and *SERPINE1* while upregulating negative regulator *TGIF1* and *SOX3*. In breast cancer cell lines, these inhibitors exhibited potent growth inhibition, with MEISi-3 showing an exceptional IC50 as low as 0.1 μM in SK-BR-3 cells. Mechanistic studies using flow cytometry revealed that these inhibitors induce dose-dependent apoptosis and necrosis. Importantly, the novel inhibitors showed minimal toxicity to healthy human dermal and MRC5 fibroblasts, suggesting a favorable safety profile. **Conclusions**: These findings establish these small molecules as promising therapeutic candidates for targeting MEIS2-dependent pathways in breast cancer.

## 1. Introduction

Homeobox transcription factors orchestrate fundamental developmental programs and are frequently dysregulated in cancer. Among them, the Three Amino Acid Loop Extension (TALE) class includes the MEIS (MEIS1-3), PBX (PBX1-4), and TGIF (TGIF1-2) families [1]. MEIS proteins in particular have emerged as crucial oncogenic drivers in solid tumors. In breast cancer, elevated *MEIS2* expression is associated with enhanced epithelial-to-mesenchymal transition (EMT), metastatic dissemination, and poor clinical outcome [2,3]. MEIS2 directly regulates a set of target genes intimately linked to invasion and stemness, including the cell adhesion molecule *CDH1* (E-cadherin), the pro-metastatic cytokine receptor *IL17RB*, the early growth response factor *EGR2*, and the developmental regulator *PAX6* [4,5]. Importantly, MEIS2 operates in reciprocal balance with the transcriptional repressor TGIF1, which can antagonize MEIS-mediated activation by competing for DNA binding or recruiting histone deacetylases [6,7]. Disruption of this MEIS2-TGIF1 equilibrium may therefore represent an attractive therapeutic strategy in MEIS-driven breast cancers.

Despite their oncogenic significance, MEIS proteins have historically been considered undruggable because they lack classical enzymatic pockets. Our group recently overcame this barrier by performing the first large-scale in silico screen against the MEIS1 homeodomain, leading to the identification of the first-in-class MEIS inhibitors MEISi-1 and MEISi-2 [8]. These compounds inhibit MEIS-dependent luciferase reporters, downregulate MEIS target genes, and reduce viability in leukemia and prostate cancer cells [9,10]. However, their efficacy in breast cancer and their ability to selectively disrupt the MEIS2-TGIF1 regulatory loop have not been explored.

In the present study, we targeted the active region of the human MEIS2 homeodomain (PDB: 3K2A) through molecular docking and identified lead candidates: the previously characterized MEISi-1 and MEISi-2 [8], together with three new inhibitors (MEISi-2E, MEISi-3, and MEISi-4) with binding energies ranging from −3.0 to −3.90 kcal/mol. We validated their inhibitory activity using a species-conserved MEIS-luciferase reporter containing the TGACAG binding motif, and we assessed their impact on the MEIS2 target gene network and the reciprocal TGIF1 axis. We demonstrate that the new-generation inhibitors potently suppress breast cancer cell growth (MEISi-3 IC_50_ as low as 0.1 µM in SK-BR-3 cells), induce dose-dependent apoptosis and necrosis, and exhibit minimal toxicity toward healthy fibroblasts. These findings establish these small molecules as promising therapeutic candidates for MEIS2-dependent breast cancer and highlight the MEIS2-TGIF1 axis as a novel pharmacological target.

## 2. Results

### 2.1. In Silico Identification and Characterization of Novel MEIS2 Inhibitors

**Figure 1:**
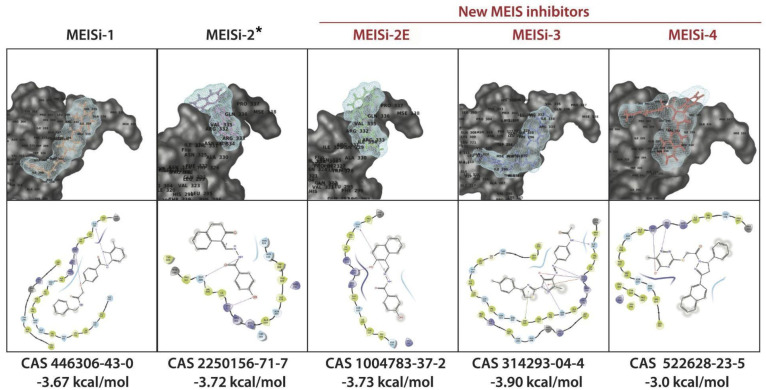
In silico docking of MEIS inhibitors. Molecular docking analysis of previously described (MEISi-1, MEISi-2) and new (MEISi-2E, MEISi-3, MEISi-4) small-molecule inhibitors into the human homeobox protein Meis2 (PDB: 3K2A). The top panels show the 3D docking poses within the protein’s active region, while the middle panels display 2D interaction diagrams highlighting binding residues. Bottom labels provide corresponding CAS numbers and binding energies (ranging from −3.0 to −3.90 kcal/mol) calculated using Maestro Glide. * MEISi-2 (Z form) included in the patent numbered EP3541410A2 was not available, thus it was not included in this study. MEISi-2 was modeled using the Z-isomer (based on ZINC301013) for consistency with previous study [8]. The commercially available material used previously was of unspecified isomeric configuration, whereas the newly synthesized compound in this study is the definitively characterized E-isomer (MEISi-2E).

To identify potent small-molecule inhibitors of the MEIS protein, we conducted a structure-based molecular docking study targeting the homeobox domain of the human Meis2 protein (PDB: 3K2A). Using the Maestro Glide package, we screened and characterized five primary candidates: MEISi-1, MEISi-2, MEISi-2E, MEISi-3, and MEISi-4. MEISi-1 and MEISi-2 have been described previously [8] and are included here as benchmark controls. MEISi-2E, MEISi-3, and MEISi-4 represent newly synthesized or newly validated inhibitors. As shown in Figure 1, these molecules were docked into the protein’s active region, with calculated binding energies ranging from −3.0 to −3.90 kcal/mol. The 3D docking poses and 2D interaction diagrams highlight specific residues within the binding pocket that stabilize the inhibitors.

Detailed 2D interaction diagrams reveal that these molecules stabilize within the homeobox binding pocket through specific residues. MEISi-1 (CAS 446306-43-0) interacts primarily with Arg 333 and Gln 336. MEISi-2 (CAS 2250156-71-7) demonstrates hydrogen bonding with Arg 333 and proximity to Gln 336. MEISi-2E (CAS 1004783-37-2) forms critical hydrogen bonds with Gln 336 and Arg 332. MEISi-3 (CAS 314293-04-4), the most potent in subsequent assays, and features multiple hydrogen bonds with Arg 333, Gln 336 and Asn 329, alongside pi-cation interactions and contact with Phe 296. MEISi-4 (CAS 522628-23-5) targets a slightly shifted pocket, forming hydrogen bonds with Arg 331 and Arg 332. While MEISi-2 was initially characterized, it was discontinued and excluded from further in vitro analysis, making MEISi-2E, MEISi-3, and MEISi-4 the primary new MEIS2 inhibitors for biological validation.

**Figure 2:**
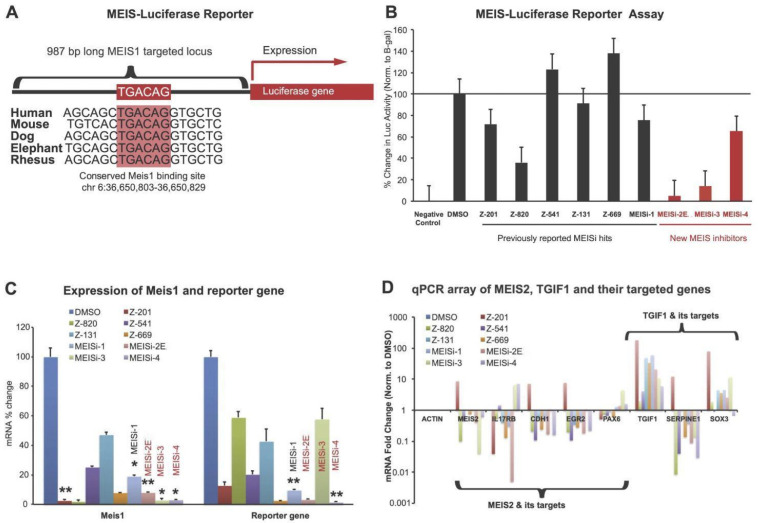
Identification and validation by in vitro luciferase reporter and gene expression. (**A**) Schematic representation of the MEIS-Luciferase Reporter construct containing a 987 bp conserved MEIS1 targeted locus (p21 conserved region) with the TGACAG binding site conserved across species. (**B**) MEIS-Luciferase Reporter Assay showing the percent change in luciferase activity (normalized to β-gal) for previously reported MEISi hits and the new inhibitors (MEISi-2E, -3, -4) compared to DMSO. (**C**) RT-qPCR analysis showing the mRNA percent change for *Meis1* and the luciferase reporter gene following inhibitor treatment. (**D**) qPCR array showing fold change in expression for *MEIS2*, *TGIF1*, and their downstream target genes (e.g., *IL17RB*, *CDH1*, *EGR2*, *PAX6*, *SERPINE1*, *SOX3*) normalized to DMSO. * *p* < 0.05, ** *p* < 0.01.

The compounds evaluated in this study fall into three categories. First, MEISi-1 and MEISi-2 are previously described first-generation inhibitors [8] included as benchmark controls. Second, five additional compounds (Z-201, Z-820, Z-541, Z-131, Z-669) were also among the top-scoring hits from the same 2020 screen but showed inferior activity in preliminary assays; they are included here only as comparative controls in the luciferase reporter assay (Figure 2B) and were not subjected to further characterization. Third, MEISi-2E, MEISi-3, and MEISi-4 represent the primary focus of this study—newly synthesized (MEISi-2E) or newly validated (MEISi-3 and MEISi-4) inhibitors. A complete listing of all compounds, including SMILES notations, CAS numbers, and molecular weights, is provided in Supplementary Table S1.

### 2.2. Synthesis and Characterization of MEISi-2E

The Schiff base derivative, MEISi-2E, was successfully synthesized via condensation of 2-hydroxy-1-naphthaldehyde with 4-hydroxybenzohydrazide in ethanol under reflux in the presence of catalytic acetic acid. The reaction proceeded smoothly, affording the desired product in an excellent yield (92%) as a crystalline solid after recrystallization from ethanol, indicating high purity and efficient imine formation. The formation of target compounds was confirmed by spectroscopic techniques (Figure S1A–C). In the ^1^H NMR spectrum (DMSO-*d*_6_), the appearance of a characteristic singlet at δ 9.45 ppm, attributable to the azomethine proton (–CH=N–), clearly indicates successful condensation (Figure S1A). The aromatic region displayed multiple signals between δ 6.89–8.16 ppm corresponding to the protons of both the naphthyl and phenyl rings, consistent with the proposed structure. The presence of phenolic hydroxyl groups was evidenced by singlets at δ 10.21 and 11.99 ppm, while the hydrazide NH proton appeared as a downfield singlet at δ 12.88 ppm, suggesting strong intramolecular hydrogen bonding.

The ^13^C NMR spectra further supports the structure, with signals in the range δ 109.01–132.90 ppm corresponding to aromatic carbons (Figure S1B). The azomethine carbon (C=N) was observed in the downfield region (typically ~δ 158–162 ppm), overlapping with signals at δ 158.29, 161.45, and 162.58 ppm, which may also include contributions from phenolic and carbonyl carbons. The presence of these deshielded carbons is consistent with the conjugated system and imine functionality.

The LC–MS spectrum showed a molecular ion peak at *m*/*z* 306.98 [M]^+^, in good agreement with the expected molecular weight of the compound, along with a sodium adduct peak at *m*/*z* 329.04 [M+Na]^+^, further confirming the molecular composition (Figure S1C).

Regarding *E/Z* isomerism, Schiff bases derived from salicylaldehyde-type systems typically favor the *E*-configuration around the C=N bond due to reduced steric hindrance and stabilization via intramolecular hydrogen bonding [11,12]. In the present compound, the presence of an ortho-hydroxyl group on the naphthyl moiety enables the formation of a strong intramolecular O–H···N hydrogen bond with the imine nitrogen, which stabilizes the *E*-isomer. Additionally, the observation of a single azomethine proton signal in the ^1^H NMR spectrum suggests the predominance of a single isomer in solution, further supporting assignment of the *E*-configuration. Of note, the previously reported MEISi-2 (commercial material, isomeric composition unspecified) and the newly synthesized MEISi-2E (definitively characterized E-isomer) are distinct compounds as the MEISi-2E is a new inhibitor introduced in this study.

Overall, the spectroscopic data are in full agreement with the proposed structure of MEISi-2E and confirm the successful formation of a highly conjugated Schiff base system, likely stabilized in the E-form through intramolecular hydrogen bonding.

### 2.3. In Vitro Validation via MEIS-Luciferase Reporter and Gene Expression Analysis

The inhibitory efficacy was first validated using a MEIS-Luciferase Reporter system. This construct features a 987 bp conserved MEIS1 targeted locus from the p21 region, containing a TGACAG binding site that is highly conserved across species, including humans, mice, dogs, and rhesus monkeys (Figure 2A). In the luciferase reporter assay, MEISi-2E, and MEISi-3 significantly suppressed luciferase activity compared to both the DMSO control and previously reported MEISi hits (Z-201, Z-820, Z-541, Z-131, and Z-669; see Supplementary Table S1) (Figure 2B), which were included as comparative controls. These Z-series compounds were among the top-scoring hits from our previously reported in silico screen [8] but were not functionally characterized in detail.

Further analysis by RT-qPCR demonstrated that these novel inhibitors markedly reduced the mRNA expression of both Meis1 and the reporter gene (Figure 2C). Specifically, MEISi-2E, MEISi-3 and MEISi-4 showed near-complete suppression of *Meis1* transcript levels and reporter gene. Additionally, a qPCR array revealed that treatment with these inhibitors modulated a broad network of genes, including the downregulation of MEIS2 as well as their downstream targets such as IL17RB, CDH1, EGR2, PAX6 [13,14,15,16,17], and upregulation of a competitive transcription factor TGIF1 and downregulation of SERPINE1 [18,19], and upregulation of SOX3 [20] (Figure 2D). This suggests that MEISi-2E, MEISi-3, and MEISi-4 could be targeting MEIS2 proteins effectively along with MEIS1.

### 2.4. Safety Profile of Novel MEIS2 Inhibitors in Healthy Cell Lines

**Figure 3:**
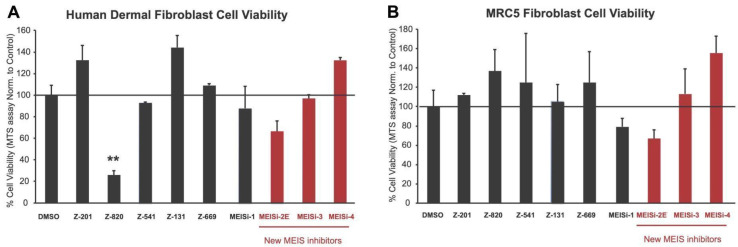
Effect of novel small-molecule MEIS2 inhibitors on viability of healthy cell lines. Cell viability analysis using the WST1 assay on (**A**) Human Dermal Fibroblast (HDF) and (**B**) MRC5 fibroblast cells after 72 h of treatment. Data are normalized to the DMSO control, indicating the safety profile of the novel inhibitors on non-cancerous cells. ** *p* < 0.01.

To evaluate the potential cytotoxic effects on non-cancerous cells, we performed WST1 viability assays on Human Dermal Fibroblasts (HDF) and MRC5 fibroblasts. Following 72 h of treatment, MEISi-2E, MEISi-3, and MEISi-4 showed no significant reduction in cell viability compared to the DMSO control, indicating a high safety threshold in these healthy cell lines (Figure 3A,B). In contrast, the previously reported hit Z-820 exhibited significant toxicity in HDF cells.

### 2.5. Growth Inhibition and IC50 Determination in MCF7 Breast Cancer Cells

**Figure 4:**
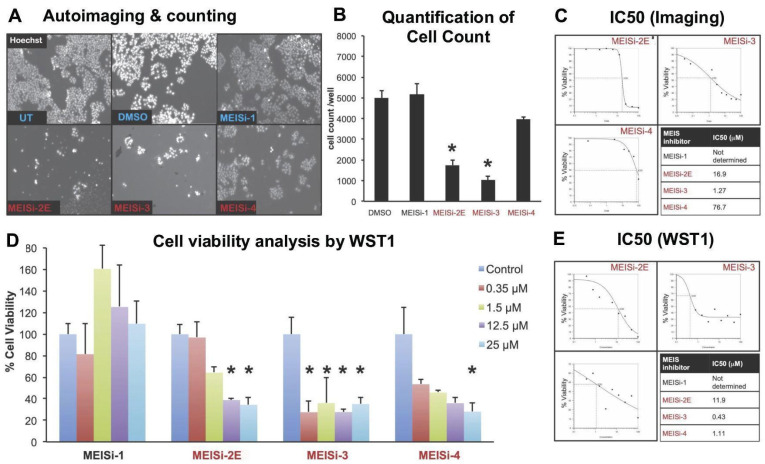
Analysis of novel small-molecule MEIS2 inhibitors in MCF7 cancer cells. (**A**) Representative Hoechst staining and autoimaging of MCF7 breast cancer cells after treatment with 25 μM of the indicated MEIS2 inhibitors. (**B**) Quantification of cell count per well based on autoimaging. (**C**) Dose–response viability curves and calculated IC50 values (μM) derived from imaging data. (**D**) WST1 cell viability analysis of MCF7 cells across a concentration range from 0.35 μM to 25 μM. (**E**) Dose–response curves and IC50 quantification based on the WST1 assay for MEISi-2E, MEISi-3, and MEISi-4. * *p* < 0.05.

The anti-cancer potential of the novel inhibitors was assessed in MCF7 breast cancer cells through imaging and viability assays. Hoechst staining and autoimaging after treatment with 25 μM of the inhibitors revealed a substantial decrease in cell density for MEISi-2E and MEISi-3 (Figure 4A). Quantification of the cell count confirmed a significant, dose-dependent reduction in cell numbers (Figure 4B). Dose–response curves generated from the image based assay (Figure 4A,B) allowed for the calculation of IC50 values (Figure 4C). MEISi-3 emerged as the most potent inhibitor in MCF7 cells with an IC50 of 1.27 μM.

In addition, we performed WST1 viability assay in four distinct doses for MEISi-1, MEISi-2E, MEISi-3, and MEISi-4. MEISi-2E treatment demonstrated significant reduction in viability as low as 12.5 μM while MEISi-3 reduced viability up to 75% as low as 0.35 μM concentration (Figure 4D). Analysis of the effect of cancer cell viability for MEIS2 inhibitors with WST1 assay allowed us to precisely determine IC50 values (Figure 4E). Among the new inhibitors, MEISi-3 emerged as the most potent inhibitor in MCF7 cells with an IC50 of 0.43 μM, followed by MEISi-4 (1.11 μM) and MEISi-2E (11.9 μM). In comparison, MEISi-1 did not reach a calculable IC50 within the tested range (Figure 4D).

### 2.6. Induction of Apoptosis and Necrosis

**Figure 5:**
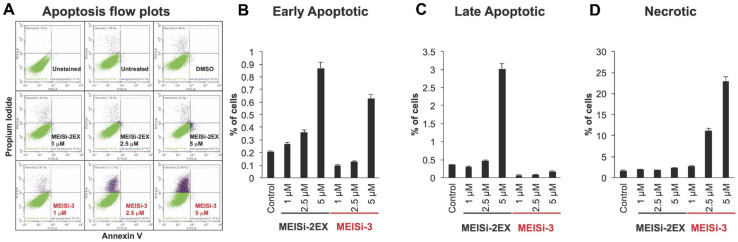
Analysis of apoptosis post small-molecule MEIS2 inhibitor treatments. (**A**) Representative flow cytometry plots using Annexin V and Propidium Iodide (PI) staining for MCF7 cells treated with varying concentrations (1, 2.5, and 5 μM) of MEISi-2E and MEISi-3. Bar graphs show the quantified percentage of (**B**) Early Apoptotic, (**C**) Late Apoptotic, and (**D**) Necrotic cells, demonstrating dose-dependent induction of cell death.

To elucidate the mode of cell death, we performed flow cytometry using Annexin V and Propidium Iodide (PI) staining on MCF7 cells treated with 1, 2.5, and 5 μM of the inhibitors (Figure 5A). MEISi-2E induced a dose-dependent increase in early and late apoptosis, peaking at 5 μM (Figure 5B,C). Conversely, MEISi-3 treatment led to a dramatic and significant increase in early apoptosis (Figure 5B) as well as necrotic cells, particularly at the 5 μM concentration, where nearly 23% of the population was necrotic (Figure 5D).

### 2.7. Potency Across Diverse Breast Cancer Cell Lines and Baseline Expression

**Figure 6:**
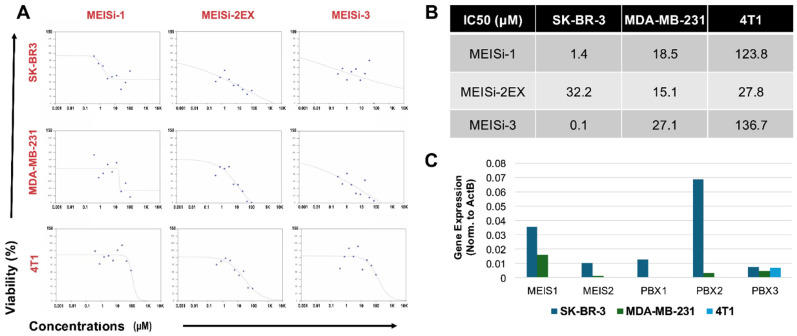
Effect of novel small-molecule MEIS2 inhibitors in other breast cancer cells. (**A**) Dose–response viability curves for SK-BR-3, MDA-MB-231, and 4T1 breast cancer cell lines treated with MEISi-1, MEISi-2E, and MEISi-3. (**B**) Table of IC50 values (μM) for each inhibitor across the three cell lines, showing high potency for MEISi-3 in SK-BR-3 cells (0.1 μM). (**C**) Baseline gene expression levels of MEIS1, MEIS2, PBX1, PBX2, and PBX3 in SK-BR-3, MDA-MB-231, and 4T1 cells, normalized to β-actin.

The broader applicability of these inhibitors was tested in SK-BR-3, MDA-MB-231, and 4T1 breast cancer cell lines (Figure 6A). MEISi-3 showed exceptional potency in SK-BR-3 cells with an IC50 of 0.1 μM, while being less effective in the more aggressive MDA-MB-231 (27.1 μM) and 4T1 (136.7 μM) lines (Figure 6B). MEISi-2E exhibited consistent efficacy across all three lines, with IC50 values ranging from 15.1 to 32.2 μM.

To investigate the basis for these varying sensitivities, we analyzed the baseline mRNA expression of MEIS1, MEIS2, and PBX1-3 (Figure 6C). The data showed that SK-BR-3 cells, which were most sensitive to MEISi-3, had the highest relative expression levels of MEIS1 and MEIS2 normalized to β-actin.

## 3. Discussion

Direct pharmacological inhibition of transcription factors has long been a formidable challenge in oncology due to their extensive protein–protein and protein–DNA interfaces and the absence of classical active-site pockets. The TALE-class homeobox protein MEIS2 drives aggressive phenotypes in breast cancer, including EMT, metastasis, and stemness, yet until recently was considered undruggable [2,4]. We previously described first-in-class small molecules targeting the MEIS1 homeodomain [8]; here, we substantially extend this conceptual advance by identifying MEISi-2E, MEISi-3, and MEISi-4 as potent second-generation inhibitors that dock into the DNA-binding pocket of the MEIS2 homeodomain and functionally block MEIS-dependent transcription. This work establishes a structural and functional framework for disrupting the MEIS1/2-TGIF1 regulatory circuit, provides a mechanistic rationale for transcription factor addiction in breast cancer subtypes, and delineates a path toward clinical translation.

MEIS2, a TALE-homeodomain transcription factor, exhibits marked context dependency in cancer, functioning as either a tumor suppressor or an oncogenic driver depending on cellular lineage and microenvironmental cues. In solid tumors such as breast and prostate cancer, reduced MEIS2 expression, often linked to promoter hypermethylation or hypoxia, has been associated with enhanced proliferation, immune evasion, and adverse clinical outcomes [21,22,23,24]. Mechanistically, MEIS2 has been implicated in restraining tumor progression through modulation of immune signaling pathways, including IL10-mediated axes in breast cancer [23]. However, this tumor-suppressive paradigm is not universal; in hematologic malignancies and selected solid tumors such as hepatocellular carcinoma, MEIS2 supports proliferation and survival via regulation of cell-cycle progression and activation of oncogenic pathways including Wnt/β-catenin and Hippo/YAP signaling [25,26]. These divergent roles underscore the necessity of context-specific interpretation when considering MEIS2 as a therapeutic target.

In this study, despite the prevailing view of MEIS2 as a tumor suppressor in breast cancer, pharmacological inhibition of MEIS2 resulted in pronounced cancer cell death in several cancer cell lines, suggesting a previously underappreciated dependency in this context. This apparent discrepancy may reflect tumor heterogeneity, context-specific rewiring of transcriptional networks, or differential isoform expression, as reported in other malignancies. Notably, evidence from multiple myeloma and AML demonstrates that MEIS2 can sustain tumor cell survival and therapeutic resistance, and that its inhibition induces apoptosis and enhances drug sensitivity [26,27]. Our findings extend this paradigm to breast cancer, indicating that, under specific molecular conditions, MEIS2 may acquire a pro-survival role amenable to therapeutic targeting. Collectively, these data support a model in which MEIS2 represents a context-dependent vulnerability and highlight the importance of biomarker-driven stratification to identify breast cancer subgroups that may benefit from MEIS2-directed therapies.

TGIF1 and TGIF2, members of the TALE superclass of atypical homeodomain proteins, display pronounced context dependency in cancer, acting as either oncogenic drivers or tumor suppressors depending on tissue and signaling context. TGIF1 is frequently overexpressed in solid tumors, including breast cancer, where it promotes proliferation, migration, and invasion, in part through direct interaction with β-catenin and activation of Wnt signaling [28,29]. TGIF2 similarly contributes to tumor progression, for example, via EGFR/ERK-dependent phosphorylation that recruits HDAC1 to repress E-cadherin and induce epithelial–mesenchymal transition in lung adenocarcinoma [30]. However, tumor-suppressive roles have also been described, as exemplified by TGIF1 in pancreatic ductal adenocarcinoma, where it restrains Twist1-driven malignancy [29]. Consistent with this functional plasticity, our data show that pharmacological inhibition of MEIS proteins leads to robust upregulation of TGIF1 in breast cancer cells, concomitant with significant induction of cell death. This suggests that, in this context, TGIF1 may participate in a tumor-suppressive transcriptional program that becomes unmasked upon disruption of MEIS-dependent signaling. Mechanistically, TGIF and MEIS proteins are known to exert opposing transcriptional functions within the TALE family. TGIF acts as a transcriptional repressor that can compete with MEIS activators for overlapping DNA-binding sites, thereby modulating gene expression in a ratio-dependent manner [31]. In hematologic malignancies, TGIF1 antagonizes MEIS1-driven oncogenic programs, and a lower MEIS1:TGIF1 ratio correlates with improved clinical outcomes [32]. Our observation that MEIS inhibition simultaneously downregulates MEIS1/MEIS2 and induces TGIF1 expression supports a model in which therapeutic targeting of MEIS shifts the balance of TALE transcriptional networks toward repression of pro-survival gene programs. This coordinated rewiring may underlie the pronounced cytotoxicity observed in breast cancer cells. While direct TGIF2-MEIS interactions remain less well defined, the shared DNA-binding specificity within the TALE family suggests that similar competitive dynamics may exist. Collectively, these findings highlight a previously underappreciated therapeutic axis in which MEIS inhibition not only suppresses oncogenic transcriptional drivers but also activates counter-regulatory repressors such as TGIF1, reinforcing cell death pathways in a context-dependent manner.

Our in silico docking positioned all active inhibitors within the conserved TGACAG-recognition helix of the MEIS2 homeodomain, engaging residues Arg332, Arg333, and Gln336 that are critical for base-specific DNA contacts [33,34]. We also observed that MEISi-3 (CAS 314293-04-4), the most potent inhibitor identified in this study, establishes additional stabilizing interactions within the HD binding pocket, including hydrogen bonds with Asn329 and Phe296, together with π–cation interactions. These interactions likely enhance binding affinity and support the feasibility of targeting the MEIS homeodomain (HD) with small-molecule inhibitors. The binding energies (−3.0 to −3.90 kcal/mol) are comparable to those of successful fragment-based leads and suggest that these molecules may competitively displace DNA. The functional suppression of the MEIS-luciferase reporter, which contains a species-conserved TGACAG motif from the *CDKN1A* locus, demonstrates that the compounds effectively neutralize MEIS transcriptional output in a cellular context. As no direct biophysical binding data (e.g., surface plasmon resonance or X-ray co-crystallography) are yet available, the precise binding mode remains to be validated. However, the strong concordance between computational prediction, reporter inhibition, and the stereoselective difference between the E-isomer (MEISi-2) and Z-isomer (MEISi-2E) provides compelling circumstantial evidence for a specific, pose-dependent interaction with the homeodomain.

A striking and unanticipated result was the profound reduction in *MEIS2* mRNA levels upon inhibitor treatment, despite the compounds being designed to block protein-DNA interactions rather than transcript synthesis. This observation points to the existence of a positive autoregulatory loop in which MEIS2 protein sustains its own expression, likely through direct binding to regulatory elements of the *MEIS2* locus. In cancer, such core transcriptional regulatory circuitries where master regulators form interconnected feed-forward loops are increasingly recognized as a mechanism that locks cells into an oncogenic state, creating vulnerability to transcriptional addiction [5]. We therefore propose that MEIS2 sustains its own expression through a comparable auto-regulatory mechanism, and that pharmacological disruption of the MEIS2 homeodomain not only blocks acute DNA binding but also triggers the collapse of this self-sustaining oncogenic circuit. This dual mechanism-immediate blockade of transcriptional activity and subsequent collapse of the MEIS2-centered transcriptome may explain the exceptional cellular potency observed for MEISi-3 (IC_50_ 0.1 µM in SK-BR-3 cells). Future nascent RNA and chromatin occupancy (ChIP-seq) experiments will be essential to delineate this auto-feedback circuit and to determine whether it represents a general vulnerability across TALE-driven cancers.

The observed downregulation of CDH1 (E-cadherin) following MEIS inhibition is consistent with a shift in TALE network balance toward TGIF-mediated transcriptional repression in breast cancer cells. Evidence in breast cancer indicates that TGIF1 expression negatively correlates with E-cadherin, suggesting a repressive function [35]. In parallel, a direct mechanistic link between TGIF2 and CDH1 silencing has been established in lung adenocarcinoma, where ERK-mediated phosphorylation of TGIF2 enables HDAC1 recruitment to the E-cadherin promoter, directly suppressing transcription and promoting epithelial–mesenchymal transition [30]. In this context, the upregulation of TGIF1 observed upon MEIS inhibition likely contributes to CDH1 repression, either directly or through cooperation with EMT-associated factors such as TWIST1. These findings suggest that MEIS inhibition not only disrupts pro-survival transcriptional programs but also induces a partial EMT-like transcriptional shift, reflected by reduced CDH1 expression; however, given the concurrent induction of cell death, this state may represent a non-productive or terminal EMT-like response rather than enhanced metastatic potential, highlighting the complex and context-dependent consequences of TALE network perturbation.

MEISi-2E and MEISi-3 triggered dose-dependent apoptosis and necrosis in MCF7 cells, indicating that MEIS pathway disruption pushes cells past a critical survival threshold. MEIS1 and MEIS2 have well-characterized roles in maintaining redox homeostasis and suppressing oxidative stress-induced apoptosis in hematopoietic stem cells and leukemias [9,36]. The concomitant appearance of necrosis, particularly at higher inhibitor concentrations, may reflect ATP depletion secondary to severe metabolic stress or direct membrane perturbation. Because MEIS proteins orchestrate a broad repertoire of metabolic and anti-apoptotic effectors, their pharmacological blockade may be particularly effective in malignancies that have become transcriptionally addicted to this network. The inability of MDA-MB-231 and 4T1 cells to undergo comparable cell death, despite measurable target modulation, underscores that transcriptional addiction to MEIS2 is not universal; these lines may possess compensatory pathways, such as alternative TALE factors or activated Wnt/β-catenin signaling, that bypass the MEIS requirement.

The exceptional sensitivity of SK-BR-3 cells (IC_50_ 0.1 µM) compared with MDA-MB-231 (27.1 µM) and 4T1 (136.7 µM) correlates tightly with the basal expression of *MEIS1* and *MEIS2* mRNA (Figure 6C). This observation extends the concept of transcription factor addiction from hematological malignancies to solid tumors. In MLL-rearranged leukemia, MEIS1 is an indispensable co-factor of HOXA9, and the two proteins cooperate to drive leukemogenesis; loss of either is synthetic lethal [37]. Similarly, HER2-amplified breast cancers may rely on MEIS2 for maintaining the transformed state, as MEIS2 has been identified among transcription factors associated with poor outcome in this subtype [38], thereby creating a potential therapeutic window. Prospective biomarker studies that measure MEIS2 protein or mRNA in patient-derived xenografts and tissue microarrays will be essential to validate this predictive relationship and to guide clinical development.

The evolution to MEISi-3 (IC_50_ 0.1 µM) represents a major improvement in potency, driven by rational design around the Arg-rich DNA-binding pocket. Notably, the profound functional difference between MEISi-2 and MEISi-2E tautomers demonstrates the stringent stereochemical requirements for productive engagement with the homeodomain and provides a critical medicinal chemistry lesson for this class of targets. Targeting a DNA-binding interface with drug-like small molecules remains rare, and the MEIS inhibitors described here add to a small but growing list of homeodomain-targeting compounds. Their selectivity against other DNA-binding proteins and the broader TALE family (PBX, PREP) has not been exhaustively profiled, but the specificity determinants within the homeodomain and the observed safety on fibroblasts provide encouragement.

The absence of significant toxicity in human dermal fibroblasts and MRC5 lung fibroblasts at concentrations far exceeding those required for cancer cell killing suggests a favorable therapeutic index, at least for mesenchymally derived normal cells. However, MEIS proteins are indispensable for normal hematopoiesis and for the proper function of cardiomyocyte-associated systems [36,39]. The observed selectivity of MEIS inhibitors for breast cancer cells over fibroblasts may arise from the differential reliance of cancer cells on high MEIS2 expression, analogous to the well-known therapeutic window for CDK4/6 inhibitors, which selectively arrest RB-proficient tumor cells while sparing RB-deficient normal tissues [40,41]. Delineating the minimal MEIS activity required for normal tissue homeostasis will be critical for establishing a safe dosing regimen.

Several important limitations must be acknowledged. MEIS proteins play essential physiological roles, including maintenance of hematopoietic stem cells [36], regulation of postnatal cardiomyocyte cell cycle arrest [39], and coordination of developmental programs [1]. Systemic inhibition of MEIS activity could therefore lead to on-target toxicities, including myelosuppression, impaired cardiac regeneration, or developmental abnormalities if administered during pregnancy. The context-dependent tumor suppressor functions of MEIS1 in certain solid tumors, such as prostate cancer, where reduced MEIS1/MEIS2 expression correlates with metastatic progression [21], raise the possibility that MEIS inhibition could theoretically promote tumor progression in specific contexts. Our observation that MEIS inhibition induces cell death in breast cancer cell lines suggests that, in this context, MEIS2 functions as an oncogenic dependency rather than a tumor suppressor. However, careful patient stratification will be essential to identify breast cancer subtypes most likely to benefit from MEIS-targeted therapies while avoiding potential deleterious effects in tissues where MEIS serves a protective role. Future in vivo studies using conditional knockout models or intermittent dosing schedules will be necessary to define the therapeutic window.

This study provides the foundational in vitro characterization of a novel chemical series, but several key gaps must be addressed to translate these findings. First, direct target engagement must be demonstrated through biophysical techniques and cellular target engagement assays (e.g., IRIS Kinetics). Second, comprehensive selectivity profiling against the TALE family, nuclear receptors, and a representative kinase panel is required to define the polypharmacology of these molecules. Third, the functional contribution of TGIF1 induction and the divergent SOX3/SERPINE1 regulation must be dissected using siRNA-mediated knockdown and TGIF1 overexpression in the presence of inhibitors, coupled with genome-wide occupancy (ChIP-seq) to map the redistribution of TGIF1 on chromatin. Fourth, genetic rescue experiments in which an inhibitor-insensitive MEIS2 mutant is introduced will be critical to establish that the observed apoptosis is on-target. Fifth, the in vivo pharmacokinetic and pharmacodynamic properties, tumor growth inhibition in orthotopic breast cancer models (including SK-BR-3 and patient-derived xenografts with high MEIS2 expression), and potential on-target toxicities must be evaluated. Finally, the mechanism of resistance should be anticipated, particularly whether compensatory upregulation of PBX or other TALE factors can bypass MEIS2 inhibition, and whether rational combination therapies (e.g., with HER2-targeted agents or CDK4/6 inhibitors) can forestall resistance.

## 4. Materials and Methods

### 4.1. In Silico Molecular Docking

Structure-based molecular docking was employed to identify and evaluate small-molecule inhibitors targeting the homeobox domain (HD) of MEIS proteins, following the overall strategy described previously [8,42]. The crystal structure of the human MEIS2 homeodomain (PDB ID: 3K2A) was retrieved from the Protein Data Bank (http://www.rcsb.org/). Water molecules, ligands, and ions were removed, and the protein was prepared using the Protein Preparation Wizard in Schrödinger Maestro (Schrödinger, LLC, New York, NY, USA). Hydrogen atoms were added, bond orders were assigned, and the structure was optimized at pH 7.0. A restrained minimization was performed to converge heavy atoms to an RMSD of 0.30 Å.

The active site grid was centered on the conserved residues known to interact with the MEIS-binding DNA motif “TGACAG” [8,34]. A grid box with dimensions sufficient to encompass the entire DNA-binding pocket was generated at coordinates x = 24.251, y = 43.841, z = 15.007 using the Glide receptor grid generation tool. This grid was used for all docking calculations.

Three-dimensional conformers of small molecules were retrieved from the ZINC database in SDF format (Table S1). For compounds existing as tautomers, the structures were manually drawn in the 2D sketcher of Maestro to generate unambiguous input geometries. All ligands were prepared using the LigPrep module with the OPLS4 force field, generating ionization states and tautomers at pH 7.0 ± 2.0, and the resulting conformers were saved in SDF format.

Molecular docking was performed using the Glide SP (Standard Precision) docking protocol [43]. Ligands were docked into the prepared grid with flexible ligand sampling. For each compound, the pose with the best GlideScore was retained. The GlideScore SP scoring function, which is an empirically based extension of ChemScore, was used to estimate binding affinity according to the formula:

GScore = 0.05 × vdW + 0.15 × Coul + Lipo + Hbond + Metal + Rewards + RotB + Site.

Protein–ligand interaction diagrams were generated to identify key contacts between the inhibitors and MEIS HD residues. The docking protocol was previously validated through enrichment analysis of known homeobox inhibitors versus random libraries, as described in our earlier work [8].

### 4.2. Compounds and Chemical Synthesis

The small molecules investigated in this study are listed in Table S1, along with their identifiers, SMILES notations, molecular weights, and CAS numbers where available. Compounds were purchased from Molport (www.molport.com). MEISi-2 (CAS 2250156-71-7) was discontinued and therefore not included in the experimental assays. To replace MEISi-2, its structural analog MEISi-2E (ZINC19227812, CAS 1004783-37-2), which shares an identical core but differs in geometry (E/Z), was synthesized by Meinox.

- Synthesis of MEISi-2E

(4-hydroxy-N-[(Z)-(2-hydroxynaphthalen-1-yl)methylideneamino]benzamide): Briefly, equimolar amounts of 2-hydroxy-1-naphthaldehyde and 4-hydroxybenzohydrazide were dissolved in ethanol in the presence of a catalytic amount of acetic acid and heated under reflux for 4 h. The reaction mixture was cooled to room temperature, and the resulting precipitate was collected by filtration, washed with cold ethanol, and dried. The crude product was purified by recrystallization from ethanol to yield MEISi-2E as a crystalline solid. The structure and purity were confirmed by ^1^H NMR, ^13^C NMR, and MS (Figure S1A–C). Stock solutions of all compounds were prepared in sterile dimethyl sulfoxide (DMSO, Calbiochem, Cat. No. 317275) at 10 mM and stored at −20 °C.

- Yield: 92%. ^1^H NMR (DMSO-*d*_6_, δppm): 6.89–6.92 (m, 2H, arH), 7.21 (d, 1H, *J* = 8.0 Hz, arH), 7.38 (t, 1H, *J* = 6.0 Hz, arH), 7.59 (t, 1H, *J* = 8.0 Hz, arH), 7.85-7.91 (m, 4H, arH), 8.16 (d, 1H, *J* = 8.0 Hz, arH), 9.45 (s, 1H, CH), 10.21 (s, 1H, OH), 11.99 (s, 1H, OH), 12.88 (s, 1H, NH). ^13^C NMR (DMSO-*d*_6_, δppm): 109.01, 115.67, 119.38, 120.89, 123.50, 123.93, 128.14, 128.22, 129.42, 130.11, 132.01, 132.90, 146.30, 158.29, 161.45, 162.58. LC-MS: 306.98 [M]^+^, 329.04 [M+Na]^+^.

### 4.3. Cell Culture

Human breast cancer cell lines MCF7, SK-BR-3, and MDA-MB-231, as well as healthy control fibroblast lines HDF and MRC5, were obtained from the American Type Culture Collection (ATCC, Manassas, VA, USA). MCF7 cells were maintained in RPMI 1640 medium (Gibco, Waltham, MA, USA, Cat. No. 31885-023) supplemented with 10% (*v*/*v*) heat-inactivated fetal bovine serum (FBS, Sigma-Aldrich, Burlington, MA, USA, Cat. No. 12103C) and 1% (*v*/*v*) penicillin–streptomycin-amphotericin B (PSA; 10,000 U/mL penicillin, 10,000 µg/mL streptomycin, 25 µg/mL amphotericin B; Gibco, Cat. No. 15240062). HDF and MRC5 cells were cultured in Dulbecco’s Modified Eagle’s Medium (DMEM) High Glucose (Gibco, Waltham, MA, USA, Cat. No. 41965-039) containing 5% (*v*/*v*) heat-inactivated FBS and 1% PSA. All cells were incubated at 37 °C in a humidified atmosphere with 5% CO_2_.

### 4.4. Cell Viability and Counting

The effect of MEIS inhibitors on cell viability was determined using the WST1 assay, as previously described [1,8]. Briefly, cells were seeded at a density of 2 × 10^4^ cells per well in 96-well plates and allowed to adhere overnight. The following day, the medium was replaced with a fresh medium containing the indicated concentrations of inhibitors (0.1–100 µM) or an equivalent volume of DMSO as vehicle control (final DMSO concentration ≤ 0.5%). After 72 h of treatment, 10 µL of WST1 solution (5 mg/mL in PBS) was added to each well, and plates were incubated for 3 h at 37 °C. The formazan crystals were dissolved in 100 µL DMSO, and absorbance was measured at 590 nm using a Varioskan LUX Multimode Microplate Reader (Thermo Fisher Scientific, Waltham, MA, USA). Cell viability was expressed as a percentage of the DMSO control. In parallel, automated cell counting was performed using the Cytell Imaging System (GE Healthcare, Chicago, IL, USA) [44]. Cells were seeded and treated as above. After 72 h, cells were stained with Hoechst 33342 (10 µg/mL; Sigma-Aldrich, Burlington, MA, USA, Cat. No. 14533) for 10 min at 37 °C. Images covering entire wells were acquired with the Cytell system, and nuclei were counted using Scion Image software (Maryland, USA). Half-maximal inhibitory concentration (IC50) values were calculated using a four-parameter logistic regression model (IC50 Calculator Tool, AAT Bioquest).

### 4.5. MEIS-Luciferase Reporter Assay

The functional inhibition of MEIS transcriptional activity was quantified using a species-conserved MEIS-luciferase reporter system as previously reported [8,36,39]. The reporter construct (p21-pGL2) contains a 987 bp genomic fragment from the p21 (CDKN1A) regulatory region encompassing the conserved MEIS-binding motif TGACAG. HEK293T cells were seeded in 6-well plates and co-transfected with 2 µg of the MEIS-Luc reporter plasmid, 400 ng of the MEIS1 expression vector pCMVSPORT6-Meis1 (Open Biosystems, Huntsville, AL, USA), and 100 ng of a β-galactosidase control plasmid (pCMV-LacZ) using polyethyleneimine transfection reagent (Santa Cruz, CA, USA, Cat. No. sc-360988A). At 24 h post-transfection, cells were treated with MEIS inhibitors at the indicated concentrations (10 µM) or DMSO (0.5%). Following an additional 48 h, cells were lysed and luciferase activity was measured using the Dual-Glo Luciferase Assay System (Promega, Madison, WI, USA, Cat. No. E2920) on a Luminoskan Ascent Microplate Luminometer (Thermo Lab System, Helsinki, Finland). Firefly luciferase activity was normalized to β-galactosidase activity to account for transfection efficiency, and results were expressed as percent inhibition relative to the DMSO control.

### 4.6. RNA Isolation and Quantitative Real-Time PCR (RT-qPCR)

Total RNA was extracted using the NucleoZOL reagent (Macherey-Nagel, North Rhine-Westphalia, Germany, Cat. No. 740404.200) following the manufacturer’s protocol. Briefly, cells were seeded at 5 × 10^5^ cells per well in 6-well plates and treated with inhibitors or DMSO for 72 h. Cells were lysed directly in the plate, and RNA was purified by phenol-chloroform extraction and precipitation. RNA concentration and purity were determined using a NanoDrop 2000 spectrophotometer (Thermo Fisher Scientific). Total RNA (2 µg) was reverse transcribed into cDNA using the iScript cDNA Synthesis Kit (Bio-Rad, Hercules, CA, USA, Cat. No. 1708890) according to the manufacturer’s instructions.

Quantitative real-time PCR was performed using SYBR Green Master Mix (Bio-Rad, Cat. No. 1725140) on a LightCycler 96 instrument (Roche, Basel, Switzerland, Cat. No. 12953). Each reaction (10 µL total) contained 20 ng of cDNA template, 0.5 µM of each forward and reverse primer, and 1× SYBR Green Master Mix. Predesigned primer pairs were obtained from PrimerBank (https://pga.mgh.harvard.edu/primerbank/ accessed on 4 July 2025) [45] and synthesized by Sentegen Biotechnology (Ankara, Türkiye). The sequences of primers used are listed in Table S2. The thermal cycling conditions were: 95 °C for 3 min, followed by 40 cycles of 95 °C for 10 s and 60 °C for 30 s. Melting curve analysis was performed to confirm amplification specificity. Gene expression levels were calculated using the ΔΔCt method, normalized to β-actin (ACTB) as the housekeeping control. Relative mRNA expression was expressed as fold change compared to DMSO-treated controls.

### 4.7. Flow Cytometry for Apoptosis Analysis

Apoptosis was assessed by dual staining with Annexin V-FITC and propidium iodide (PI), using the Annexin V-FITC Apoptosis Detection Kit (Invitrogen, Waltham, MA, USA, Cat. No. BMS500FI-20) according to the manufacturer’s instructions with slight modifications [9]. Cells were seeded at 2 × 10^5^ cells/well in 6-well plates, allowed to adhere overnight, and then treated with inhibitors (10 µM) or DMSO for 72 h. Both floating and adherent cells were harvested by trypsinization and centrifugation at 300× *g* for 5 min. The cell pellet was washed once with cold PBS and resuspended in 50 µL of 1× Annexin-binding buffer. Then, 1 µL of Annexin V-FITC and 1 µL of PI (20 µg/mL) were added to each sample, gently mixed, and incubated for 15 min at room temperature in the dark. After incubation, 200 µL of 1× binding buffer was added, and samples were immediately analyzed on a CytoFlex S Flow Cytometer (Beckman Coulter, Indianapolis, IN, USA, Cat. No. B47903). Data was processed using CytExpert software 2.6.

### 4.8. Statistical Analysis

Data are presented as the mean ± standard error. Statistical significance was determined using Student’s *t*-test. *p*-values less than 0.05 were considered statistically significant (*, *p* < 0.05; **, *p* < 0.01).

### 4.9. Use of AI Tools

The authors utilized artificial intelligence-assisted tools, including NotebookLM (Google) and DeepSeek (DeepSeek-V3), to support literature organization, manuscript drafting and editing, and the development of the graphical abstract. All AI-generated outputs were critically reviewed, revised, and validated by the authors, who take full responsibility for the accuracy, integrity, and content of the final manuscript.

## 5. Conclusions

In summary, we have discovered new small molecules (MEISi-2E, MEISi-3, and MEISi-4) that disrupt DNA-binding function, suppress an auto-regulatory oncogenic loop, rewire the MEIS1/2-TGIF1 transcriptional network, and induce robust apoptosis in MEIS-addicted breast cancer cells with minimal fibroblast toxicity. These findings define the MEIS2 homeodomain as a tractable drug target, provide a mechanistic framework for transcription factor addiction in breast cancer, and lay the groundwork for the development of a new class of anti-cancer agents. Therapeutically exploiting the MEIS2 dependency uncovered here will require a concerted multidisciplinary effort spanning medicinal chemistry, target validation, and biomarker-driven clinical development.

## Data Availability

The original contributions presented in this study are included in the article/Supplementary Materials. Further inquiries can be directed to the corresponding author.

## Supplementary Materials

The following supporting information can be downloaded at: https://www.mdpi.com/article/10.3390/ph19060881/s1.
