# Supplementary material for: Synthesis and Development of Novel Small-Molecule MEIS2 Inhibitors That Induce Cell Death in Breast Cancer Cells by Targeting the Homeobox Domain
Source: Pharmaceuticals (Basel). 2026 Jun 1;19(6):881. doi: 10.3390/ph19060881 (PMC13306155; doi:10.3390/ph19060881)
Supplement: Supplementary file 1 [file pharmaceuticals-19-00881-s001.zip › pharmaceuticals-4318836-supplementary.pdf]

Supplementary Files

Supplementary Figures and Figure Legends

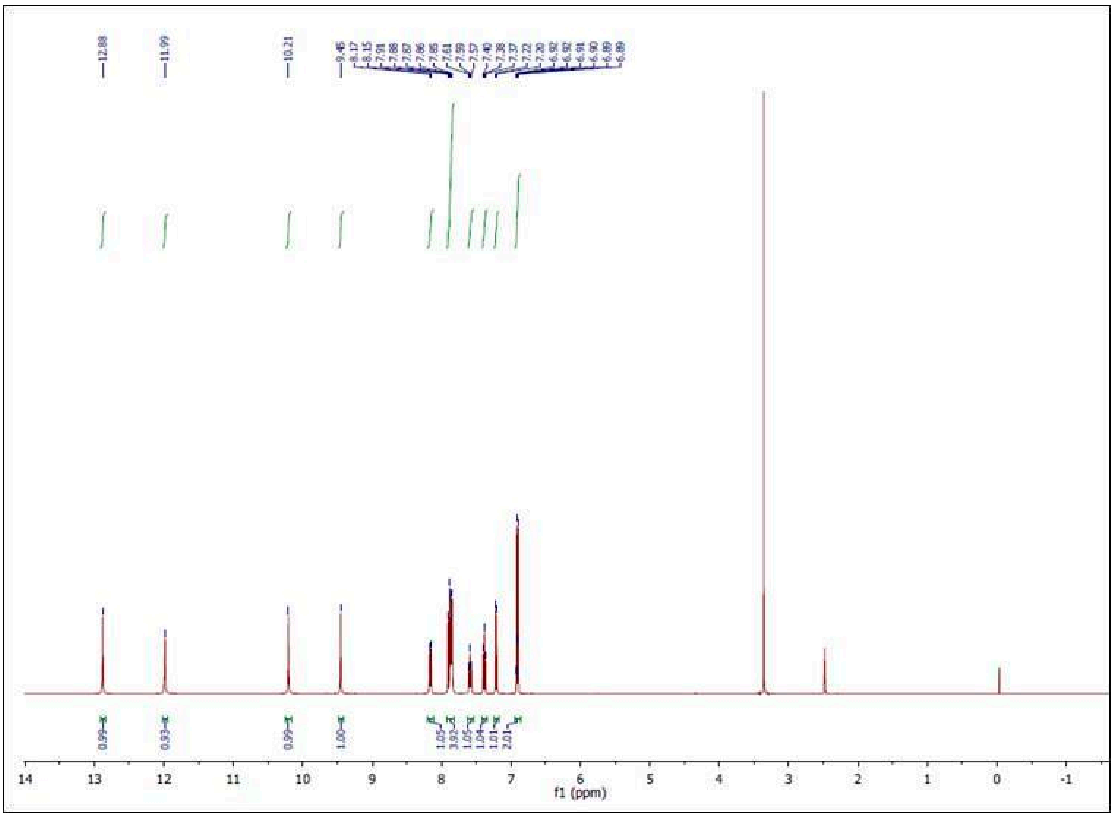

Figure S1A. ^{1}H NMR spectra of MEISi-2E

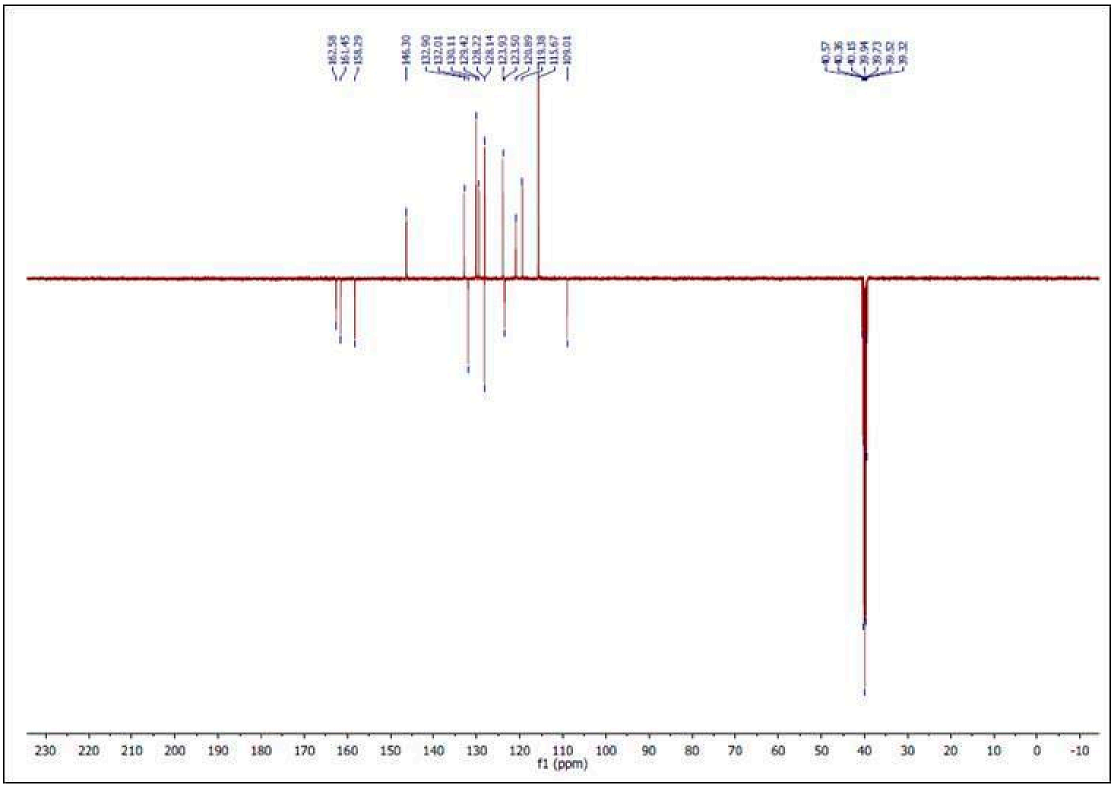

Figure S1B. ^{13}C **NMR spectra of MEISi-2E**

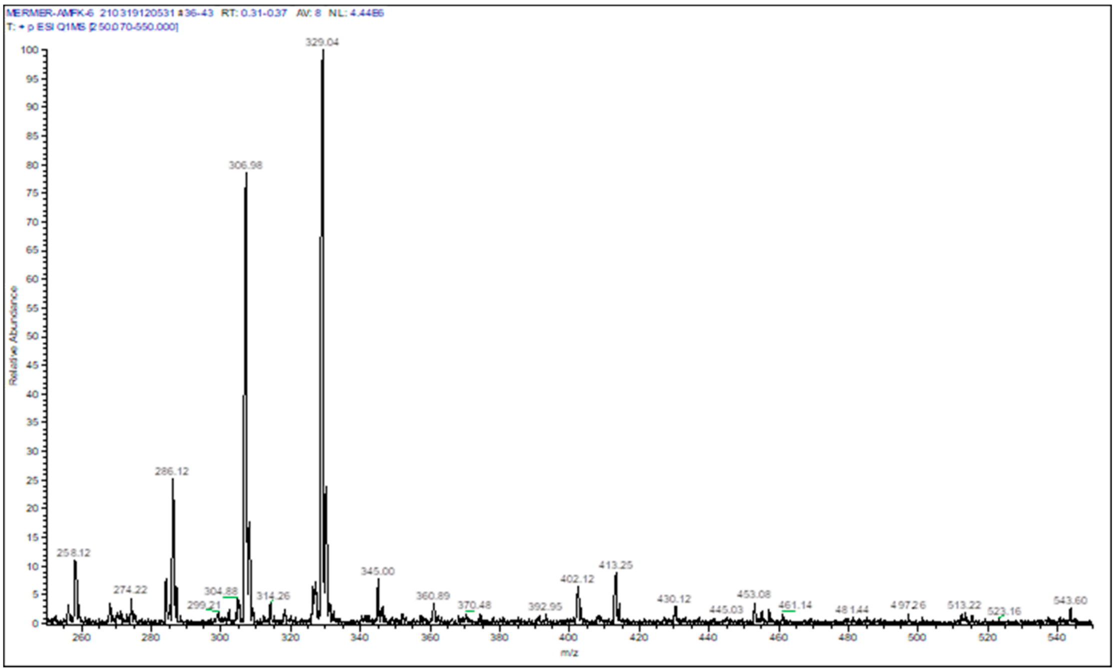

Figure S1C. LC-MS spectra of MEISi-2E

Supplementary Tables

Table S1. List of small molecules tested in this study.

ID	ZINC ID	SMILES	IUPAC Name	MW	CAS No.
**Z-201**	ZINC 9425201	C[C@H]1Cc2cc(C(=O)C3=C(O)C(=O)N(CCc4ccccc4)[C@@H]3c3ccc([N+](=O)[O-])cc3)ccc2O1	(5R)-4-[hydroxy-[(2S)-2-methyl-2,3-dihydro-1-benzofuran-5-yl]methylidene]-5-(4-nitrophenyl)-1-(2-phenylethyl)pyrrolidine-2,3-dione	484.508	NA
**Z-820**	ZINC 49406820	O=C(NC1CCN(C(=O)c2cc(-c3cccc(Cl)c3)n[nH]2)CC1)c1ccc(F)c(F)c1	N-[1-[3-(3-chlorophenyl)-1H-pyrazole-5-carbonyl]piperidin-4-yl]-3,4-difluorobenzamide	444.869	NA
**Z-541**	ZINC 71853541	O=C(Cc1csc(-c2ccccc2)n1)N1CCCN(c2nc3ccccc3[nH]2)CC1	1-[4-(1H-benzimidazol-2-yl)-1,4-diazepan-1-yl]-2-(2-phenyl-1,3-thiazol-4-yl)ethanone	417.538	CAS 1289317-09-4
**Z-131**	ZINC 00807131	Cc1ccc(NC(=O)Cn2cnc3c(cnn3-c3ccc(C)c(C)c3)c2=O)c(C)c1	N-(2,4-dimethylphenyl)-2-[1-(3,4-dimethylphenyl)-4-oxopyrazolo[5,4-d]pyrimidin-5-yl]acetamide	401.47	CAS 841211-86-7
**Z-669**	ZINC 06647669	O=C(Nc1ccc2c(c1)CCC2)c1ccc2c(c1)C[C@H](c1ccccc1)OC2=O	(3S)-N-(2,3-dihydro-1H-inden-5-yl)-1-oxo-3-phenyl-3,4-dihydroisochromene-6-carboxamide	383.447	CAS 871797-97-6
**MEISi-1**	ZINC 954985	Cc1cccc(NC(=O)c2ccc(OCC(=O)NCc3ccccc3)cc2)c1C	4-[2-(benzylamino)-2-oxoethoxy]-N-(2,3-dimethylphenyl)benzamide	388.467	CAS 446306-43-0
**MEISi-2***	ZINC 301013	O=C(N/N=C/c1c(O)ccc2ccccc12)c1ccc(O)cc1	4-hydroxy-N-[(Z)-(2-hydroxynaphthalen-1-yl)methylideneamino]benzamide	306.321	CAS 2250156-71-7
**MEISi-2E**	ZINC 19227	O=C(N/N=C\c1c(O)ccc2ccccc12)c1ccc(O)cc1	4-hydroxy-N-[(E)-(2-hydroxynaphthalen-1-	306.321	CAS 1004783-37-2

	812		yl)methylideneamino]benzamide		
**MEISi-3**	ZINC 13896 602	CC(=O)Nc1ccc(N/N=C2\C(=O)N(c3nc(-c4ccc(C)cc4)cs3)N=C2C)cc1	N-[4-[[5-methyl-2-[4-(4-methylphenyl)-1,3-thiazol-2-yl]-3-oxo-1H-pyrazol-4-yl]diazenyl]phenyl]acetamide	432.509	CAS 314293-04-4
**MEISi-4**	ZINC 94427 25	Cc1n[nH]c(SCC(=O)N2N=C(c3ccc4ccccc4c3)C[C@H]2c2ccccc2)nc1=O	6-methyl-3-[2-[(3S)-5-naphthalen-2-yl-3-phenyl-3,4-dihydropyrazol-2-yl]-2-oxoethyl]sulfanyl-4H-1,2,4-triazin-5-one	455.543	CAS 522628-23-5

*MEISi-2 (Z form) included in the patent numbered EP3541410A2 was not available, thus it was not included in this study. The commercially available compound (CAS 2250156-71-7) was supplied without stereochemical assignment. The SMILES shown correspond to the Z-isomer from ZINC301013.

Table S2. List of primer sequences used in this study

Gene ID	Forward Primer (5`->3`)	Reverse Primer (5`->3`)	Description / Purpose
**MEIS1**	GGGCATGGATGGAGTAGGC	GGGTACTGATGCGAGTGCAG	Primary target gene and baseline expression analysis.
**p21**	TGCGTTCACAGGTGTTTCTG	GTCCACTGGGCCGAAGAG	Reporter gene / Downstream target of MEIS
**MEIS2**	GAAAAGGTCCACGAACTGTGC	CTTTCATCAATGACGAGGTCGAT	Primary target gene and baseline expression analysis.
**IL17RB**	GGCTGCCTAGACCACATAATG	GCTGTGTTGGATAAGAGCCAT	Downstream target gene of MEIS2
**CDH1**	AAAGGCCCATTTCCTAAAAACCT	TGCGTTCTCTATCCAGAGGCT	Downstream target gene of MEIS2
**EGR2**	GACACGGCACATCCGAATC	GCACTGCTTTTCCGCTCTTT	Downstream target gene of MEIS2
**PAX6**	AACGATAACATACCAAGCGTGT	GGTCTGCCCGTTCAACATC	Downstream target gene of MEIS2
**TGIF1**	GGGATTGGCTGTATGAGCACC	GGCGGGAAATTGTGAACTGA	TALE family member
**SERPINE1**	AGTGGACTTTTCAGAGGTGGA	GCCGTTGAAGTAGAGGGCATT	Downstream target gene of TGIF1
**SOX3**	ACCAGGACCGTGTGAAACG	CGTCGATGAATGGTCGCTTCT	Downstream target gene of TGIF1

**PBX1**	CATGCTGTTAGCGGAAGGC	CTCCACTGAGTTGTCTGAACC	TALE family member
**PBX2**	ATGGACGAACGGCTACTGG	CCCCGATGTCTTGCTTCCC	TALE family member
**PBX3**	ATTACAGAGCCAAATTGACCCAG	TCTCGGAGAAGGTTCATCACAT	TALE family member
**β-Actin**	ATGGAGGGGAATACAGCCC	TTCTTTGCAGCTCCTTCGTT	Housekeeping control used to normalize gene expression.
